# Current State of the Clinical Applications of Artificial Intelligence in Stroke: A Literature Review

**DOI:** 10.3390/brainsci16020173

**Published:** 2026-01-31

**Authors:** Grant C. Sorkin, Nicholas M. Caffes, John P. Shank, James L. Hershey, Dana E. Knaub, Jillian C. Krebs, Muhammad H. Niazi

**Affiliations:** Division of Neurological Surgery, WellSpan Health, York, PA 17402, USA

**Keywords:** artificial intelligence, machine learning, deep learning, stroke, telestroke, clinical application, phase of care, stroke care continuum, telerehabilitation

## Abstract

Background: Artificial intelligence (AI) has emerged as a transformative tool in medicine, leveraging rapid analysis of large datasets to accelerate diagnosis, enhance clinical decision-making, and improve clinical workflows. This is highly relevant in stroke care given the time-sensitive nature of the disease process. This review evaluates the current landscape of evidence-based medicine utilizing AI in stroke, with emphasis on its use in phases of clinical care across the stroke continuum, including pre-hospital, acute, and recovery phases. This offers a comprehensive understanding of the current state of AI in both practice and literature. Methods: A review of major databases was conducted, identifying peer-reviewed literature evaluating the use of AI and its level of evidence across the stroke continuum. Given the heterogeneity of study designs, interventions, and outcome metrics spanning multiple disciplines, findings were synthesized narratively. Results: Across all phases of care, there remain no randomized controlled trials (RCTs) evaluating patient-level outcome data using AI (Level A). In the pre-hospital phase of care, AI has been used to identify stroke symptoms and assist EMS routing/training but presently remains limited to research. AI is most studied in the acute phase of care, representing the only phase to achieve commercial application in imaging detection and telestroke assistance, supported by non-randomized evidence (Level B-NR). In the recovery phase, AI may enhance wearable technologies, tele-rehabilitation, and robotics/brain–computer interfaces, with early RCTs (Level B-R) supporting the latter two, representing the strongest evidence for AI in stroke care to date. Conclusions: Despite the potential for AI to transform all phases of care across the stroke continuum, major challenges remain, including transparency, generalizability, equity, and the need for externally validated clinical studies.

## 1. Introduction

Stroke is a leading cause of morbidity and mortality, with improved outcomes associated with expedited diagnosis and treatment [1]. It affects more than 12 million people annually, represents one of the most time-sensitive emergencies in modern healthcare, and starts with primary prevention through risk factor modification and public education [2]. Current strategies for management include time-sensitive identification of large vessel occlusions (LVOs) using advanced imaging techniques, followed by vessel recanalization using systemic thrombolytics and intra-arterial thrombectomy therapies [3,4,5,6,7,8]. The delivery of these time-sensitive treatments becomes more difficult across geographically and socioeconomically diverse healthcare systems spanning urban, suburban, and rural regions, with uneven access to medical resources and advanced therapies [9,10].

Artificial intelligence has become increasingly integrated in healthcare, with strong appeal to stroke applications given the need for rapid diagnosis and treatment [11,12,13,14]. Artificial intelligence, an umbrella term incorporating machine learning, neural networks, and deep learning, may accelerate the diagnosis and treatment through interpreting and standardizing imaging, optimizing triage and transfer workflows, and risk stratification with predictive analytics, thereby narrowing geographic and socioeconomic inequalities [15,16,17,18,19]. Conceptually, machine learning is a broad field where computers use data models to learn patterns from human-constructed datasets. When connected, they create neural networks that consist of layers of data with increasing volume and complexity [20]. Deep learning models further connect neural networks into multiple layers, creating datasets so large and complex, with exponentially increasing computational power, that computers recognize patterns independent of human input or identification [20]. The choice of computational models becomes contingent on the complexity of the study question [20]. The presence of intracranial hemorrhage on non-contrast computer tomography might be identified with machine learning models while predictive analytics for stroke outcome and recurrence might require more complex deep learning models [21,22,23,24,25,26].

The existing literature evaluates AI used for stroke, categorized by specific applications including diagnostic imaging/interpretation, predictive analytics, workflow optimizations, and rehabilitation domains. This covers a broad array of topics whose audience spans multiple disciplines that participate directly or indirectly in the care of stroke patients, ranging from clinicians, to researchers, to engineers, to government-sponsored and private industry. This creates a piecemeal understanding of AI in stroke, weighted heavily to technical data and computational application, rather than a more general perspective of its clinical application and body of evidence. This review evaluates the current landscape of evidence-based medicine by organizing AI used in stroke care into clinical phases across the stroke continuum, including pre-hospital, acute, and recovery phases. By evaluating the literature according to clinical phase rather than application function, we hope to achieve a more global understanding of AI’s current implementation and remaining gaps in evidence.

## 2. Methodology

We conducted a narrative literature review of peer-reviewed published research from 2015 to 2025. Search terms included “artificial intelligence,” “machine learning,” “deep learning,” “neural network,” “stroke,” “community health systems,” “rural,” “telemedicine,” “telestroke,” “pre-hospital,” “stroke recovery,” and “stroke rehabilitation.” Databases including PubMed, Ovid Medline, Embase, and Scopus were searched using “or” as a Boolean operator restricted to title field and abstract, while using medical subject headings and synonym expansion. Inclusion criteria were peer-reviewed articles identified through the search strategy, including systematic, scoping, and narrative reviews; meta-analyses; validation and device trials; expert opinion articles; and original research with small sample sizes. Exclusion criteria included non-peer-reviewed papers, preprints, bench science without clinical relevance, and conference abstracts without full papers. Study selection was determined by agreement of the senior authors (GCS, MHN). Because the studies included were heterogeneous in designs, interventions, and outcomes, spanning multiple disciplines in a rapidly evolving, industry-dependent field, we used a narrative synthesis to provide a global view of the literature, organizing evidence into phases of clinical care across the stroke continuum with level of evidence according to the American College of Cardiology/American Heart Association classification system [27]. Given this review is secondary research of publicly available literature without patient identifiers, it is exempt from local institutional review board approval.

## 3. Results

### 3.1. Summary of Types of Existing Literature on AI Used for Stroke Care Across All Phases

The present body of literature evaluating AI for stroke is largely composed of retrospective studies evaluating proof-of-concept algorithms for imaging detecting, patient-level triage, and predictive analytics [13,21,22,23,24,25,26,28,29,30,31,32,33,34,35,36,37,38,39,40,41,42,43,44,45,46,47,48,49,50,51,52,53,54,55,56,57,58,59,60,61,62,63,64,65,66,67,68,69,70,71,72,73,74,75]. This is followed by systematic, scoping, and narrative literature reviews of AI’s specific application in stroke care [11,14,15,16,17,18,76,77,78]. There remains a paucity of prospective studies with no randomized control trials evaluating patient-level outcomes [13,79]. Applying the American College of Cardiology/American Heart Association level of evidence classification system, the body of existing literature is largely composed of Level B-NR, and Level C studies with no Level A and few Level B-R randomized controlled trails [27]. Please refer to Level of Evidence Table 1, Table 2 and Table 3, which are categorized into phases of clinical care.

### 3.2. Summary of AI Use in Pre-Hospital Phase of Care

AI is increasingly applied to pre-hospital workflows to minimize delays in the recognition and treatment of acute ischemic stroke [76,77,78]. It has the potential to enhance the recognition of symptomatology by the patient as well as enhancing paramedic evaluation and triage in facilitating transfer to thrombectomy centers [82,126]. AI tools currently being evaluated in this phase of care can be broadly categorized into stroke recognition tools, Emergency Medical Services (EMS) tools, and novel strategies, all of which are in early stages of evaluation rather than commercially available [12]. Stroke recognition tools include wearable devices like watches, fitness bands, and rings to assess motor and gait characteristics, as well as smart tablets/devices and televisions that incorporate AI recognition of facial and speech patterns to identify stroke symptomatology [83,90,91,92,127]. EMS tools currently being evaluated use AI for (1) speech pattern recognition to assist EMS dispatchers, (2) optimizing ground and air ambulance routing for more efficient activation, and (3) enhancement of personnel training and competency [80,81,84,86,87,88,89,128]. Novel strategies of noninvasive and portable versions of EEGs, TCDs, or headsets that study electrical impedance of brain tissue remain at early stages of study, while telemedicine-enabled ambulances using AI-assisted recognition software demonstrate potential to overcome the limitations of scalability of mobile stroke units [85,129,130,131]. While such tools demonstrate feasibility and promise in their application, more research is required to achieve commercial availability.

### 3.3. Summary of AI Use in the Acute Phase of Care

The acute phase of care, defined as the index hospitalization for a stroke event encompassing emergency, perioperative, and postoperative care through discharge, is the most extensively represented phase in the current AI stroke literature [11,14,15,16,17,18,77,78]. The progress made in this domain has resulted in several commercial platforms currently available on national and global scales, having achieved 510 (k) or full Food and Drug Administration clearance, as of this paper [132,133,134,135,136,137,138]. These AI applications largely apply to advanced stroke imaging acquisition, automation, and interpretation, which can be customized to healthcare systems depending on stroke program infrastructure. These include LVO detection on computer tomography angiography, computer tomography perfusion mapping, magnetic resonance imaging diffuse weight imaging analysis, automated intracranial hemorrhage detection and classification, and Alberta Stroke Program Early CT Score automation [14,15,16,17,77,78,79,98,99,100,101,102,103,104,105,106,107,108]. Because these tools promote faster diagnosis by AI interpretation and imaging cue prioritization, they have the potential to streamline workflows and reduce both medical and surgical treatment delays [93,94,95,109]. They also have potential to improve emergency department stroke workflows and help bridge gaps in specialty coverage in systems lacking neurology or neuroradiology [14,15,16,17,18,77,78,95]. This phase of care derives the most benefit from AI-supported telemedicine platforms that allow remote neurologists to triage patients by immediate imaging interpretation, independent of radiology workflows, thereby reducing time to medical and revascularization treatment [94,95,96,97,110,111]. These platforms now incorporate physician communication capabilities which show promise in improving stroke alert activation time, tele-neurology activation time, and neurointerventional consultation times [96]. Lastly, this phase of care has the highest concentration of AI use for predictive analytics in stroke care [18]. These analytic tools have been used to assess functional outcomes after stroke [28,29,30,31,32], reperfusion therapy outcomes [33,34,35], stroke etiology and risk recurrence [36,37,38,39,40,41,42,43,62,63,64,65,66], extracranial carotid artery risk factors [21,44,45,46], and comorbidity complication outcomes including pneumonia and acute kidney injury [67,68,69,70].

### 3.4. Summary of AI Use in the Recovery Phase of Care

There has been rapid increase in the body of literature describing the use of AI for stroke rehabilitation over the last 10 years, with most studies being small, single-center and pilot feasibility studies using robotic integrated AI [117,119]. Motor applications include wearable sensors, AI-integrated robotics and exoskeletons that provide adaptive services, and AI virtual reality environments that can be customized to offer patient specific recovery experiences [112,117,119,121,124,125]. Neurocognitive applications include AI-driven apps with personalized adaptive algorithms and natural language processing-based analyses for speech, cognitive, and executive function training [120,122,123]. These tools, in combination with AI-enabled wearable technology that provides real-time feedback and predictive analytics, can help facilitate remote tele-rehabilitation services [112,117,119,121,124,125,139,140,141]. Brain–computer interfaces represent a growing area of investigation in stroke care, translating electrical, magnetic, or metabolic brain activity into control signals for external devices capable of replacing, restoring, or supplementing neurological function. Their use may serve as an adjunct to both motor rehabilitation and assistance in stroke patients for acute and chronic neurological deficits [113,114,115,116,118]. In the current literature, AI use in tele-rehabilitation and brain–computer interfaces represent the strongest evidence for AI in stroke care across clinical phases, with multiple small randomized controlled trials achieving Level B-R evidence [114,122,123]. While such tools demonstrate feasibility and early benefit in their application, more research is required to demonstrate benefit and achieve commercial availability.

## 4. Discussion

In the paradigm of therapy for LVOs, healthcare delivery required realignment to rapidly identify stroke, followed by vessel recanalization using systemic and intra-arterial therapies [3,4,5,6,7,8]. AI represents another realignment in healthcare delivery, offering accelerated diagnosis, predictive analytics, and treatment. These are synergistic with goals of time-dependent stroke workflows. By structuring the literature according to phases of stroke care, our review provides a more holistic understanding of AI’s application in clinical practice.

**Figure 1:**
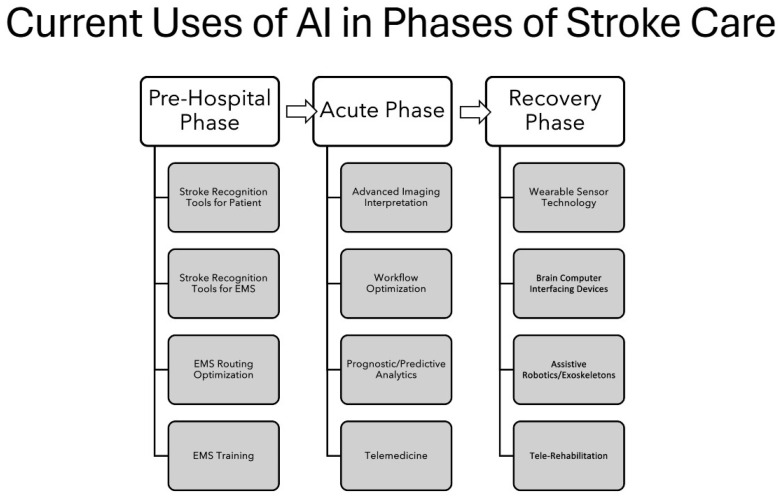
Current state of AI applications in phases of stroke care. Arrows indicate progression of clinical care through the stroke continuum.

Existing reviews of AI in stroke care are narrowly focused, most often addressing AI-assisted LVO detection or workflow augmentation to expedite revascularization, with limited attention beyond the acute phase of stroke management [11,14,15,16,17,18,142]. This is demonstrated by the larger body of evidence in the acute phase (Table 2), compared to the pre-hospital (Table 1) or recovery phases (Table 3). Stroke care is a spectrum that encompasses a continuous, coordinated process beginning with primary prevention through risk factor modification and public education, extending through long-term recovery and, when appropriate, palliative care. Once stroke occurs, rapid pre-hospital recognition and triage are critical to ensure timely transport to stroke centers capable of providing diagnostic and revascularization therapies. The remaining acute care then becomes dedicated to etiologic evaluation, prevention of early complications, and initiation of secondary prevention strategies using antithrombic therapy for cardiovascular risk factor optimization. Rehabilitation then becomes an integral component of care, transitioning from the acute to recovery phase, emphasizing functional recovery through multidisciplinary therapy in addressing intermediate and long-term needs. Organizing the literature by these phases, as illustrated in Figure 1, offers a clearer, more clinically relevant view of current AI applications in stroke care.

The acute phase of care is the most represented in the current body of literature, with a concentration in imaging detection and interpretation, telemedicine and workflow augmentation, and predictive analytics. Despite consisting of non-randomized, Level B evidence (Level B-NR), to our knowledge, this is the only phase of care that has reached commercial application and scalability. However, the pre-hospital and recovery phases of care remain as important as the acute phase at the patient-level and should offer similar potential for research, innovation, and industry-sponsored collaboration. This is supported by several prospective RCTs that provide the strongest evidence to date for AI in the recovery phase of care, specifically evaluating its use in tele-rehabilitation [122,123] and brain–computer interfaces (Level B-R) [114].

Despite its immense potential, substantial challenges with AI integration in stroke care remain. Commercial availability of AI-enabled stroke tools in the acute phase of care and widespread clinical adoption should not be interpreted as evidence of proven patient-level benefit. Several platforms have achieved regulatory approval and become integrated into clinical workflows; however, randomized controlled trials and Level A evidence demonstrating improvement in functional outcomes or mortality remain lacking and no governing society has incorporated AI into stroke guideline statements to date [143]. Accordingly, the current evidence primarily supports operational and workflow efficiencies rather than definitive clinical efficacy. These are dependent on phase of care, local infrastructure, and institutional resources, thereby limiting broad standardization. Variability in data quality, imaging protocols, and industry vendors can affect algorithm performance and generalizability, particularly when tools are deployed outside of the environment in which they were developed. This compounds concern regarding algorithm bias and transparency [11,15,17,19]. There also remain ethical and regulatory questions about data equity, ownership, and protection, which, in summation, hinders trust among end users and results in hesitancy of clinician adoption [11,15,17,19]. Lastly, while AI’s use can narrow a geographic divide, care must be taken to prevent the widening of socioeconomic disparities in infrastructure involving availability of broadband or cloud computing resources, availability of neurology, neuroradiology or neurointerventional specialties, availability of telemedicine resources, availably of on-site information technology resources, and availability of electronic health record resources [10,144]. These components require significant capital expenditure without dedicated reimbursement, thereby contributing to delay in adoption by smaller or rural healthcare systems [10,144].

## 5. Limitations of This Review

This is a narrative synthesis rather than a full systematic review with defined search strings and independent double screening. While this was chosen to accommodate a broad scope of literature that spanned disciplines, topics, and outcomes, it can increase susceptibility to selection bias and incomplete coverage of relevant studies. The absence of formal risk-of-bias assessments, and reproducible methodology for the literature search, study selection, and data extraction limits the ability to critically appraise the quality of included studies, which is particularly important in AI research where heterogeneity in datasets, algorithms, and validation methods can be common. These limitations reduce transparency and make it difficult to draw robust conclusions about clinical effectiveness or generalizability. While narrative reviews are valuable for providing conceptual overviews and identifying emerging themes in a rapidly evolving field such as AI in stroke, they offer weaker evidentiary support than systematic reviews when informing clinical practice or policy decisions. Lastly, given the field’s rapidly evolving nature, driven in part by industry-sponsored collaboration, new high-quality trials, registries, and proprietary data continue to emerge, thereby creating an expiration date on a review discussing current state.

## 6. Conclusions

Spanning early symptom recognition by patients and EMS, rapid identification and treatment of large vessel occlusions, and AI-supported tele-rehabilitation and robotics, AI is positioned to impact all phases of stroke care. While key challenges remain regarding transparency, generalizability, and external validation, AI will continue to play an important role in healthcare realignment.

## Figures and Tables

**Table 1:** Pre-hospital-phase level of evidence [27].

Ref #	Author	Date	Study Design	Topic	Level of Evidence
[80]	Ceklic E, et al.	2022	Retrospective observational cohort study	EMS routing optimization	B-NR
[81]	Rava RA, et al.	2021	Retrospective modeling study	EMS routing optimization	B-NR
[82]	Ramos A, et al.	2021	Narrative review	EMS stroke recognition	C-EO
[83]	Wasselius J, et al.	2021	Prospective diagnostic study	EMS stoke recognition	B-NR
[84]	Scholz ML, et al.	2022	Retrospective modeling study	EMS stroke recognition	B-NR
[85]	Van Stigt MN, et al.	2013	Prospective diagnostic study	EMS stroke recognition	B-NR
[86]	Caceres JA, et al.	2013	Retrospective observational cohort study	EMS stroke recognition	B-NR
[87]	Oostema JA, et al.	2018	Retrospective observational study	EMS stroke recognition	B-NR
[88]	Jia J, et al.	2017	Retrospective observational study	EMS stroke recognition	B-NR
[89]	Pottle, J	2019	Narrative review	EMS training	C-EO
[12]	Wollcott ZC, et al.	2024	Expert commentary	EMS workflow optimization	C-EO
[90]	Bat-Erdine BO, et al.	2021	Narrative review	Patient stroke recognition	C-EO
[91]	Kim HS, et al.	2015	Observational diagnostic accuracy study	Patient stroke recognition	C-LD
[92]	Raychey RI, et al.	2023	Proof of concept study	Patient stroke recognition	C-LD

**Table 2:** Acute-phase level of evidence [27].

Ref #	Author	Year	Study Design	Topic	Level of Evidence
[93]	Figurell ME, et al.	2023	Retrospective workflow implementation study	Workflow optimization	B-NR
[94]	Gunda B, et al.	2022	Retrospective workflow implementation study	Workflow optimization	B-NR
[95]	Field NC, et al.	2023	Retrospective workflow implementation study	Workflow optimization	B-NR
[96]	Devlin T, et al.	2024	Retrospective multicenter cohort study	Workflow optimization	B-NR
[97]	Hassan AE, et al.	2022	Retrospective observational study	Workflow optimization	B-NR
[31]	Herzog L, et al.	2023	Prospective prognostic model study	Prognostication	B-NR
[33]	Zhang H, et al.	2024	Retrospective prognostic model study	Prognostication	B-NR
[34]	Velagapudi L, et al.	2021	Retrospective prognostic model study	Prognostication	B-NR
[35]	Wang X, et al.	2024	Retrospective prognostic model study	Prognostication	B-NR
[32]	Liu Y, et al.	2024	Retrospective predictive model study	Prognostication	B-NR
[28]	van Os HJA, et al.	2018	Registry prognostic model study	Prognostication	B-NR
[29]	Heo J, et al.	2019	Retrospective prognostic model study	Prognostication	B-NR
[30]	Jung HS, et al.	2024	Prospective prognostic model study	Prognostication	B-NR
[11]	Koska IO, et al.	2023	Narrative review	Advanced imaging interpretation	C-EO
[32]	Liu Y, et al.	2024	Narrative review	Advanced imaging interpretation	C-EO
[14]	Chandrabhatla AS, et al.	2023	Narrative review	Advanced imaging interpretation	C-EO
[64]	Ryu WS, et al.	2024	Retrospective diagnostic model study	Advanced imaging interpretation	B-NR
[66]	Christiansen SD, et al.	2024	Retrospective diagnostic model study	Advanced imaging interpretation	B-NR
[79]	Savage CH, et al	2024	Prospective observational diagnostic study	Advanced imaging interpretation	B-NR
[98]	Olive-Gadea M, et al.	2020	Retrospective diagnostic imaging study	Advanced imaging interpretation	B-NR
[99]	McLouth J, et al.	2021	Retrospective diagnostic imaging study	Advanced imaging interpretation	B-NR
[100]	Yahav-Dovrat A, et al.	2021	Retrospective diagnostic imaging study	Advanced imaging interpretation	B-NR
[101]	Schlossman J, et al.	2022	Retrospective diagnostic imaging study	Advanced imaging interpretation	B-NR
[102]	Luijten SPR, et al.	2022	Retrospective diagnostic imaging study	Advanced imaging interpretation	B-NR
[103]	Cimflova P, et al.	2022	Retrospective diagnostic imaging study	Advanced imaging interpretation	B-NR
[104]	Bathla G, et al.	2022	Retrospective diagnostic imaging study	Advanced imaging interpretation	B-NR
[105]	Adamou A, et al.	2023	Systematic review	Advanced imaging interpretation	C-EO
[106]	Wolff L, et al.	2021	Retrospective diagnostic imaging study	Advanced imaging interpretation	B-NR
[107]	Lee SJ, et al.	2024	Prospective diagnostic imaging study	Advanced imaging interpretation	B-NR
[108]	Jiang B, et al.	2025	Systematic review	Advanced imaging interpretation	C-EO
[109]	Alwood BT, et al.	2024	Multicenter retrospective diagnostic study	Advanced imaging interpretation	B-NR
[15]	Wang Z, et al.	2024	Systematic review	General	C-EO
[16]	Melo S, et al.	2025	Scoping review	General	C-EO
[17]	Heeralal VT, et al.	2025	Narrative review	General	C-EO
[18]	Heo J.	2025	Scoping review	General	C-EO
[36]	Colangelo G, et al.	2024	Retrospective predictive model study	Predictive analytics	B-NR
[37]	Gao Y, et al.	2024	Retrospective predictive model study	Predictive analytics	B-NR
[38]	Lip GYH, et al.	2024	Retrospective predictive model study	Predictive analytics	B-NR
[39]	Vodencarevic A, et al.	2022	Retrospective predictive model study	Predictive analytics	B-NR
[40]	Han L, et al.	2019	Retrospective predictive model study	Predictive analytics	B-NR
[41]	Chen B, et al.	2022	Retrospective predictive model study	Predictive analytics	B-NR
[42]	Chao CJ, et al.	2022	Retrospective predictive model study	Predictive analytics	B-NR
[43]	Lin L, et al.	2024	Retrospective predictive model study	Predictive analytics	B-NR
[44]	Araki T, et al.	2017	Retrospective predictive model study	Predictive analytics	B-NR
[45]	Bai P, et al.	2020	Retrospective predictive model study	Predictive analytics	B-NR
[46]	Sue SS, et al.	2023	Retrospective predictive model study	Predictive analytics	B-NR
[21]	Chen YF, et al.	2023	Retrospective predictive model study	Predictive analytics	B-NR
[63]	Rabinstein AA, et al.	2021	Retrospective predictive model study	Predictive analytics	B-NR
[65]	Heo J, et al.	2023	Retrospective multicenter diagnostic model study	Prognostication	B-NR
[67]	Li X, et al.	2020	Retrospective diagnostic model study	Prognostication	B-NR
[69]	Lu X, et al.	2024	Retrospective diagnostic/prognostic model study	Prognostication	B-NR
[70]	Heo J, et al.	2022	Retrospective prognostic model study	Prognostication	B-NR
[110]	Ali F, et al.	2020	Narrative review	Telemedicine optimization	C-EO
[111]	Mohamed A, et al.	2023	Systematic review and meta-analysis	Telemedicine optimization	C-EO

**Table 3:** Recovery-phase level of evidence [27].

Ref #	Author	Year	Study design	Topic	Level of Evidence
[112]	Vélez-Guerrero MA, et al.	2021	Narrative review	Assistive robotics/exoskeletons	C-EO
[113]	Várkuti B, et al.	2012	Prospective interventional cohort	Brain-computer interfacing devices	B-NR
[114]	Cervera MA, et al.	2018	Meta analysis of RCTs	Brain-computer interfacing devices	C-EO
[115]	Bundy DT, et al.	2017	Prospective interventional cohort study	Brain-computer interfacing devices	B-NR
[116]	Mrachacz-Kersting N, et al.	2016	Prospective interventional cohort study	Brain-computer interfacing devices	B-NR
[117]	Senadheera I, et al.	2024	Scoping review	General	C-EO
[118]	Buetefisch CM	2015	Narrative review	General	C-EO
[119]	Dobkin A	2024	Scoping review	General	C-EO
[120]	Choo YJ, et al.	2022	Narrative review	General	C-EO
[121]	Sale P, et al.	2018	Prospective observational study	General	B-NR
[122]	Kim S, et al.	2025	Multicenter RCT	Tele-rehabilitation	B-R
[123]	Liscano Y, et al.	2025	Systematic review and meta-analysis	Tele-rehabilitation	B-R
[124]	Kim W, et al.	2016	Prospective observational validation study	Wearable sensor technology	B-NR
[125]	Shull PB, et al.	2019	Prospective sensor validation study	Wearable sensor technology	B-NR

## Data Availability

No new data were created or analyzed in this study.

## Abbreviations

AI	Artificial Intelligence
RCTs	Randomized Controlled Trials
LVO	Large Vessel Occlusion
EMS	Emergency Medical Services
EEG	Electroencephalogram
TCD	Transcranial Doppler

## References

[1] Saver J.L. (2006). Time is Brain-quantified. Stroke.

[2] Tsao C.W., Aday A.W., Almarzooq Z.I., Anderson C.A.M., Arora P., Avery C.L., Baker-Smith C.M., Beaton A.Z., Boehme A.K., Buxton A.E. (2023). Heart disease and stroke statistics—2023 update: A report from the American Heart Association. Circulation.

[3] Berkhemer O.A., Fransen P.S.S., Beumer D., van den Berg L.A., Lingsma H.F., Yoo A.J., Schonewille W.J., Vos J.A., Nederkoorn P.J., Wermer M.J.H. (2015). A randomized trial of intraarterial treatment for acute ischemic stroke. N. Engl. J. Med..

[4] Goyal M., Demchuk A.M., Menon B.K., Eesa M., Rempel J.L., Thornton J., Roy D., Jovin T.G., Willinsky R.A., Sapkota B.L. (2015). Randomized assessment of rapid endovascular treatment of ischemic stroke. N. Engl. J. Med..

[5] Saver J.L., Goyal M., Bonafe A., Diener H.C., Levy E.I., Pereira V.M., Albers G.W., Cognard C., Cohen D.J., Hacke W. (2015). Stent-retriever thrombectomy after intravenous t-PA vs. t-PA alone in stroke. N. Engl. J. Med..

[6] Campbell B.C.V., Mitchell P.J., Kleinig T.J., Dewey H.M., Churilov L., Yassi N., Yan B., Dowling R.J., Parsons M.W., Oxley T.J. (2015). Endovascular therapy for ischemic stroke with perfusion-imaging selection. N. Engl. J. Med..

[7] Jovin T.G., Chamorro A., Cobo E., de Miquel M.A., Molina C.A., Rovira A., San Román L., Serena J., Abilleira S., Ribó M. (2015). Thrombectomy within 8 hours after symptom onset in ischemic stroke. N. Engl. J. Med..

[8] Goyal M., Menon B.K., van Zwam W.H., Dippel D.W.J., Mitchell P.J., Demchuk A.M., Dávalos A., Majoie C.B.L.M., van der Lugt A., de Miquel M.A. (2016). Endovascular thrombectomy after large-vessel ischaemic stroke: A meta-analysis of individual patient data from five randomised trials. Lancet.

[9] Ikeme S., Kottenmeier E., Uzochukwu G., Albright K.C., Brinjiki W. (2022). Evidence-Based Disparities in Stroke Care Metrics and Outcomes in the United States: A Systematic Review. Stroke.

[10] Pantoja-Ruiz C., Akinyemi R., Lucumi-Cuesta D.I., Owolabi M.O., Pandian J.D., Strong K., Feigin V.L., Bennett D.A., O’Donnell M.J., Mensah G.A. (2025). Socioeconomic status and stroke: A review of the latest evidence on inequalities and their drivers. Stroke.

[11] Koska I.O., Selver A. (2023). Artificial intelligence in stroke imaging: A comprehensive review. Eurasian J. Med..

[12] Wolcott Z.C., English S.W. (2024). Artificial intelligence to enhance prehospital stroke diagnosis and triage: A perspective. Front. Neurol..

[13] Karamian A., Seifi A. (2025). Diagnostic accuracy of deep learning for intracranial hemorrhage detection in noncontrast brain CT scans: A systematic review and meta-analysis. J. Clin. Med..

[14] Chandrabhatla A.S., Kuo E.A., Sokolowski J.D., Kellogg R.T., Park M., Mastorakos P. (2023). Artificial intelligence and machine learning in the diagnosis and management of stroke: A narrative review of United States Food and Drug Administration-approved technologies. J. Clin. Med..

[15] Wang Z., Yang W., Li Z., Rong Z., Wang X., Han J., Ma L. (2024). A 25-year retrospective of artificial intelligence for diagnosing acute stroke. J. Med. Internet Res..

[16] Melo Sierra N., Hernández Rincón E.H., Osorio Betancourt G.A., Ramos Chaparro P.A., Diaz Quijano D.M., Barbosa S.D., Hernandez Restrepo M., Uriza Sinisterra G. (2025). Use of artificial intelligence in the management of stroke: Scoping review. Front. Radiol..

[17] Heeralal V.T., Chadee S.E., Ilyaev B., Ilyaev R., Ilyayeva S. (2025). Artificial intelligence in stroke care: A narrative review of diagnostic, predictive, and workflow applications. Cureus.

[18] Heo J. (2025). Application of artificial intelligence in acute ischemic stroke: A scoping review. Neurointervention.

[19] Feng S., Song S., He T. (2025). Application and challenges of artificial intelligence in the care of stroke patients. J. Clin. Nurs. Res..

[20] LeCun Y., Bengio Y., Hinton G. (2015). Deep learning. Nature.

[21] Chen Y.F., Chen Z.J., Lin Y.Y., Lin Z.Q., Chen C.N., Yang M.L., Zhang J.Y., Li Y.Z., Wang Y., Huang Y.H. (2023). Stroke risk study based on deep learning-based magnetic resonance imaging carotid plaque automatic segmentation algorithm. Front. Cardiovasc. Med..

[22] Li H., Gao M., Song H., Wu X., Li G., Cui Y., Zhang Z., Liu D., Zhao S., Chen J. (2023). Predicting ischemic stroke risk from atrial fibrillation based on multi-spectral fundus images using deep learning. Front. Cardiovasc. Med..

[23] Messica S., Presil D., Hoch Y., Lev T., Hadad A., Katz O., Cohen O., Lunt S., Gilead I., Tsfadia Y. (2024). Enhancing stroke risk and prognostic timeframe assessment with deep learning and a broad range of retinal biomarkers. Artif. Intell. Med..

[24] Wei J., Shang K., Wei X., Zhu Y., Yuan Y., Wang M., Li S., Zhang L., Chen Y., Liu Q. (2025). Deep learning-based automatic ASPECTS calculation can improve diagnosis efficiency in patients with acute ischemic stroke: A multicenter study. Eur. Radiol..

[25] Ostmeier S., Axelrod B., Liu Y., Yu Y., Jiang B., Yuen N., Rao V.M., Mittal S., Shuaib A., Figueiredo G. (2025). Random expert sampling for deep learning segmentation of acute ischemic stroke on non-contrast CT. J. NeuroInterventional Surg..

[26] Mohapatra S., Lee T.H., Sahoo P.K., Wu C.Y. (2023). Localization of early infarction on non-contrast CT images in acute ischemic stroke with deep learning approach. Sci. Rep..

[27] Otto C.M., Abdullah A.R., Smith S.C., Jones D.S., Patel M.R., Benjamin E.J., Bhatt D.L., Giri J., Heidenreich P.A., Lloyd-Jones D.M. (2025). 2025 ACC/AHA guideline core principles and development process: A report of the American College of Cardiology/American Heart Association Joint Committee on Clinical Practice Guidelines. J. Am. Coll. Cardiol..

[28] van Os H.J.A., Ramos L.A., Hilbert A., van Leeuwen M., van Walderveen M.A.A., Kruyt N.D., Dippel D.W.J., Steyerberg E.W., van der Schaaf I.C., Lingsma H.F. (2018). Predicting outcome of endovascular treatment for acute ischemic stroke: Potential value of machine learning algorithms. Front. Neurol..

[29] Heo J., Yoon J.G., Park H., Kim Y.D., Nam H.S., Heo J.H. (2019). Machine learning-based model for prediction of outcomes in acute stroke. Stroke.

[30] Jung H.-S., Lee E.-J., Chang D.-I., Cho H.J., Lee J., Cha J.-K., Park M.-S., Yu K.H., Jung J.-M., Ahn S.H. (2024). A multimodal ensemble deep learning model for functional outcome prognosis of stroke patients. J. Stroke.

[31] Herzog L., Kook L., Hamann J., Globas C., Heldner M.R., Seiffge D., Antonenko K., Dobrocky T., Panos L., Kaesmacher J. (2023). Deep learning versus neurologists: Functional outcome prediction in LVO stroke patients undergoing mechanical thrombectomy. Stroke.

[32] Liu Y., Shah P., Yu Y., Horsey J., Ouyang J., Jiang B., Yang G., Heit J.J., McCullough-Hicks M.E., Hugdal S.M. (2024). A clinical and imaging fused deep learning model matches expert clinician prediction of 90-day stroke outcomes. Am. J. Neuroradiol..

[33] Zhang H., Polson J.S., Wang Z., Nael K., Rao N.M., Speier W.F., Yeung V., Kazim S.F., Liu X., Ali A. (2024). A deep learning approach to predict recanalization first-pass effect following mechanical thrombectomy in patients with acute ischemic stroke. Am. J. Neuroradiol..

[34] Velagapudi L., Mouchtouris N., Schmidt R.F., Vuong D., Khanna O., Sweid A., Hasan T.F., Ospel J.M., Mehta B.P., Tjoumakaris S.I. (2021). A machine learning approach to first pass reperfusion in mechanical thrombectomy: Prediction and feature analysis. J. Stroke Cerebrovasc. Dis..

[35] Wang X., Luo S., Cui X., Qu H., Zhao Y., Liao Q. (2024). Machine learning-based predictive model for the development of thrombolysis resistance in patients with acute ischemic stroke. BMC Neurol..

[36] Colangelo G., Ribó M., Montiel E., Dominguez D., Olivé-Gadea M., Muchada M., Quintana M., Poca M.A., Dávalos A., Boada I. (2024). PRERISK: A personalized, artificial intelligence-based and statistically-based stroke recurrence predictor for recurrent stroke. Stroke.

[37] Gao Y., Li Z.A., Zhai X.Y., Han L., Zhang P., Cheng S.J., Wang Y., Xu Y., Song J., Li H. (2024). An interpretable machine learning model for stroke recurrence in patients with symptomatic intracranial atherosclerotic arterial stenosis. Front. Neurosci..

[38] Lip G.Y.H., Genaidy A., Tran G., Marroquin P., Estes C., Sloop S. (2022). Improving stroke risk prediction in the general population: A comparative assessment of common clinical rules, a new multimorbid index, and machine-learning-based algorithms. Thromb. Haemost..

[39] Vodencarevic A., Weingärtner M., Caro J.J., Ukalovic D., Zimmermann-Rittereiser M., Schwab S., Paul F., Heller E., Koch P., Heuschmann P.U. (2022). Prediction of recurrent ischemic stroke using registry data and machine learning methods: The Erlangen Stroke Registry. Stroke.

[40] Han L., Askari M., Altman R.B., Schmitt S.K., Fan J., Bentley J.P., Zhang S., Neubeck L., Wang X., Zou J. (2019). Atrial fibrillation burden signature and near-term prediction of stroke: A machine learning analysis. Circ. Cardiovasc. Qual. Outcomes.

[41] Chen B., Ruan L., Yang L., Zhang Y., Lu Y., Sang Y., Guo J., Huang Z., Liu X., Wang K. (2022). Machine learning improves risk stratification of coronary heart disease and stroke. Ann. Transl. Med..

[42] Chao C.J., Agasthi P., Barry T., Chiang C.C., Wang P., Ashraf H., Siddiqui T., Mamas M.A., McCrae K., Vemulapalli S. (2023). Using artificial intelligence in predicting ischemic stroke events after percutaneous coronary intervention. J. Invasive Cardiol..

[43] Lin L., Ding L., Fu Z., Zhang L. (2024). Machine learning-based models for prediction of the risk of stroke in coronary artery disease patients receiving coronary revascularization. PLoS ONE.

[44] Araki T., Jain P.K., Suri H.S., Londhe N.D., Ikeda N., El-Baz A., Shrivastava V.K., Saba L., Nicolaides A., Shafique S. (2017). Stroke risk stratification and its validation using ultrasonic echolucent carotid wall plaque morphology: A machine learning paradigm. Comput. Biol. Med..

[45] Bai P., Zhou Y., Liu Y., Li G., Li Z., Wang T., Wei X., Zhang L., Qiu X., Feng W. (2020). Risk factors of cerebral infarction and myocardial infarction after carotid endarterectomy analyzed by machine learning. Comput. Math. Methods Med..

[46] Su S.S., Li L.Y., Wang Y., Li Y.Z. (2023). Stroke risk prediction by color Doppler ultrasound of carotid artery-based deep learning using Inception V3 and VGG-16. Front. Neurol..

[47] Rudnicka A.R., Welikala R., Barman S., Foster P.J., Luben R., Hayat S., Khaw K.T., Whincup P., Strachan D., Owen C.G. (2022). Artificial intelligence-enabled retinal vasculometry for prediction of circulatory mortality, myocardial infarction and stroke. Br. J. Ophthalmol..

[48] Guberina N., Dietrich U., Radbruch A., Goebel J., Deuschl C., Ringelstein A., Kornhuber M., Buerger K., Heiland S., Sure U. (2018). Detection of early infarction signs with machine learning-based diagnosis by means of the Alberta Stroke Program Early CT score (ASPECTS) in the clinical routine. Neuroradiology.

[49] Hoelter P., Muehlen I., Goelitz P., Beuscher V., Schwab S., Doerfler A. (2020). Automated ASPECT scoring in acute ischemic stroke: Comparison of three software tools. Neuroradiology.

[50] Delio P.R., Wong M.L., Tsai J.P., Hinson H.E., McMenamy J., Le T.Q., Ospel J.M., Mehta B.P., Lindsay P., Hill M.D. (2021). Assistance from automated ASPECTS software improves reader performance. J. Stroke Cerebrovasc. Dis..

[51] Matsoukas S., Morey J., Lock G., Chada D., Shigematsu T., Marayati N.F., Munoz C., Hess C., Bonafé A., Jovin T.G. (2023). AI software detection of large vessel occlusion stroke on CT angiography: A real-world prospective diagnostic test accuracy study. J. NeuroInterventional Surg..

[52] Dehkharghani S., Lansberg M.G., Venkatsubramanian C., Cereda C., Lima F.O., Coelho H., Mlynash M., Kim A.S., Olivot J.M., Bammer R. (2021). High-performance automated anterior circulation CT angiographic clot detection in acute stroke: A multireader comparison. Radiology.

[53] Grunwald I.Q., Kulikovski J., Reith W., Gerry S., Namias R., Politi M., Lynch J.R., Sommer W.H., Meyer L., Stolz E. (2019). Collateral automation for triage in stroke: Evaluating automated scoring of collaterals in acute stroke on computed tomography scans. Cerebrovasc. Dis..

[54] Czap A.L., Bahr-Hosseini M., Singh N., Yamal J.M., Nour M., Parker S., Gupta R., Nesbit G.M., Peterson E.C., Fruth O. (2022). Machine learning automated detection of large vessel occlusion from mobile stroke unit computed tomography angiography. Stroke.

[55] Qiu W., Kuang H., Teleg E., Ospel J.M., Sohn S.I., Almekhlafi M.A., Coutts S.B., Hill M.D., Demchuk A.M., Goyal M. (2020). Machine learning for detecting early infarction in acute stroke with non-contrast-enhanced CT. Radiology.

[56] Nishi H., Ishii A., Tsuji H., Fuchigami T., Sasaki N., Tachibana A., Yokote K., Ohara S., Sakai K., Uemura J. (2023). Automatic ischemic core estimation based on noncontrast-enhanced computed tomography. Stroke.

[57] Altmann S., Grauhan N.F., Brockstedt L., Kondova M., Schmidtmann I., Paul R., Dinkel J., Fritz J., Lobsien D., Shah J. (2024). Ultrafast brain MRI with deep learning reconstruction for suspected acute ischemic stroke. Radiology.

[58] You S.H., Cho Y., Kim B., Yang K.S., Kim I., Kim B.K., Im S.H., Park H.M., Lee S.J., Seo D.W. (2023). Deep learning-based synthetic TOF-MRA generation using time-resolved MRA in fast stroke imaging. Am. J. Neuroradiol..

[59] Wenstrup J., Havtorn J.D., Borgholt L., Blomberg S.N., Maaloe L., Sayre M.R., Deal M., Lee H.K., Persson O., Lauritsen S.M. (2023). A retrospective study on machine learning-assisted stroke recognition for medical helpline calls. npj Digit. Med..

[60] Park E., Chang H.J., Nam H.S. (2017). Use of machine learning classifiers and sensor data to detect neurological deficit in stroke patients. J. Med. Internet Res..

[61] Ou Z., Wang H., Zhang B., Liang H., Hu B., Ren L., Li X., Chen Y., Zhou T., Yang L. (2025). Early identification of stroke through deep learning with multi-modal human speech and movement data. Neural Regen. Res..

[62] Kamel H., Navi B.B., Parikh N.S., Merkler A.E., Okin P.M., Devereux R.B., Elkind M.S.V., Iadecola C., Healey J.S., Wintermark M. (2020). Machine learning prediction of stroke mechanism in embolic strokes of undetermined source. Stroke.

[63] Rabinstein A.A., Yost M.D., Faust L., Kashou A.H., Latif O.S., Graff-Radford J., Siegler J.E., Weingart J.D., Lopes D.K., Mesfin F.B. (2021). Artificial intelligence-enabled ECG to identify silent atrial fibrillation in embolic stroke of unknown source. J. Stroke Cerebrovasc. Dis..

[64] Ryu W.S., Schellingerhout D., Lee H., Lee K.J., Kim C.K., Kim B.J., Kwon S.U., Hong K.S., Heo T.W., Kim H.C. (2024). Deep learning-based automatic classification of ischemic stroke subtype using diffusion-weighted images. J. Stroke.

[65] Heo J., Lee H., Seog Y., Kim S., Baek J.H., Park H., Lee D.H., Yoon B.H., Sohn S.I., Nam H.S. (2023). Cancer prediction with machine learning of thrombi from thrombectomy in stroke: Multicenter development and validation. Stroke.

[66] Christiansen S.D., Liu J., Bullrich M.B., Sharma M., Boulton M., Pandey S.K., Cheloth N.D., Saini V., Khatri P., Demchuk A.M. (2024). Deep learning prediction of stroke thrombus red blood cell content from multiparametric MRI. Interv. Neuroradiol..

[67] Li X., Wu M., Sun C., Zhao Z., Wang F., Zheng X., Chen Y., Liu J., Zhang H., Wang L. (2020). Using machine learning to predict stroke-associated pneumonia in Chinese acute ischemic stroke patients. Eur. J. Neurol..

[68] Lee C.C., Su S.Y., Sung S.F. (2024). Machine learning-based survival analysis approaches for predicting the risk of pneumonia post-stroke discharge. Int. J. Med. Inf..

[69] Lu X., Chen Y., Zhang G., Zeng X., Lai L., Qu C. (2024). Application of interpretable machine learning algorithms to predict acute kidney injury in patients with cerebral infarction in ICU. J. Stroke Cerebrovasc. Dis..

[70] Heo J., Yoo J., Lee H., Lee I.H., Kim J.S., Park E., Nam H.S., Kim Y.D., Hong K.S., Sohn S.I. (2022). Prediction of hidden coronary artery disease using machine learning in patients with acute ischemic stroke. Neurology.

[71] Hassan A., Benlamri R., Diner T., Cristofaro K., Dillistone L., Khallouki H., Gauthier S., Dubeau C., Singh B., Hachinski V. (2024). An app for navigating patient transportation and acute stroke care in Northwestern Ontario using machine learning: Retrospective study. JMIR Form. Res..

[72] Lim D.Z., Yeo M., Dahan A., Tahayori B., Kok H.K., Abbasi-Rad M., Ng J., Lim Z.Y., Wong Z.R., Tan J.Y. (2022). Development of a machine learning-based real-time location system to streamline acute endovascular intervention in acute stroke: A proof-of-concept study. J. NeuroInterventional Surg..

[73] Chiramal J.A., Johnson J., Webster J., Nag D.R., Robert D., Ghosh T., Golla S., Pawar S., Krishnan P., Drain P.K. (2024). Artificial intelligence-based automated CT brain interpretation can improve uptake of lifesaving interventions in resource-limited settings. PLoS Glob. Public Health.

[74] Agripnidis T., Ayobi A., Quenet S., Beland B., Smith D., Liu Y., Chen L., Roberts J., Patel M., Johnson K. (2025). Performance of an artificial intelligence tool for multi-step acute stroke imaging: A multicenter diagnostic study. Eur. J. Radiol. Open.

[75] Leonardsen A.C.L., Hardeland C., Dehre A., Vik E., Jensen T., Hansen R., Pedersen L., Olsson C., Nilsson A., Larsson K. (2025). Emergency medical services providers’ perspectives on the use of artificial intelligence in prehospital identification of stroke: A qualitative study in Norway and Sweden. BMC Emerg. Med..

[76] Perez K., Wisniewski D., Ari A., Johnson T., Smith P., Li J., Patel R., Nguyen M., Chen L., Brown A. (2025). Investigation into application of AI and telemedicine in rural communities: A systematic literature review. Healthcare.

[77] Mouridsen K., Thurner P., Zaharchuk G. (2020). Artificial intelligence applications in stroke. Stroke.

[78] Liu Y., Wen Z., Wang Y., Chen L., Zhang H., Li X., Zhao F., Yang J., Xu T., Huang Q. (2024). Artificial intelligence in ischemic stroke images: Current applications and future directions. Front. Neurol..

[79] Savage C.H., Tanwar M., Abou Elkassem A., Patel P., Li Y., Wong T., Chen R., Smith J., Brown M., Garcia L. (2024). Prospective evaluation of artificial intelligence triage of intracranial hemorrhage on noncontrast head CT examinations. AJR Am. J. Roentgenol..

[80] Ceklic E., Ball S., Finn J., Brown E., Brink D., Bailey P., Smith K., Cameron P., Fitzgerald M., Walker T. (2022). Ambulance dispatch prioritisation for traffic crashes using machine learning: A natural language approach. Int. J. Med. Inform..

[81] Rava R.A., Peterson E.D., Hastrup S., Mocco J., Smith W.S., Saver J.L., Liebeskind D.S., Jahan R., Jovin T.G., Nogueira R.G. (2021). AI routing on mobile stroke units. Stroke.

[82] Ramos A., Guerrero W.R., Pérez De La Ossa N. (2021). Prehospital stroke triage. Neurology.

[83] Wasselius J., Finn E.L., Persson E., Song G., Wu S., Feng S., Lindgren A., Norrving B., Engström G., Svensson P. (2021). Detection of unilateral arm paresis after stroke by wearable accelerometers and machine learning. Sensors.

[84] Scholz M.L., Collatz-Christensen H., Blomberg S.N.F., Boebel S., Verhoeven J., Krafft T. (2022). Artificial intelligence in emergency medical services dispatching: Assessing the potential impact of an automatic speech recognition software on stroke detection taking the Capital Region of Denmark as case in point. Scand. J. Trauma Resusc. Emerg. Med..

[85] Van Stigt M.N., Groenendijk E.A., Van Meenen L.C.C., van de Munckhof A., Coutinho J.M., de Maat M.P.M., Kerkhof F., van der Worp H.B., Roos Y.B.W.E.M., Dippel D.W.J. (2023). Prehospital detection of large vessel occlusion stroke with EEG: Results of the ELECTRA-STROKE study. Neurology.

[86] Caceres J.A., Adil M.M., Jadhav V., Chaudhry S.A., Pawar S., Rodriguez G.J., Suri M.F.K., Qureshi A.I. (2013). Diagnosis of stroke by emergency medical dispatchers and its impact on the prehospital care of patients. J. Stroke Cerebrovasc. Dis..

[87] Oostema J.A., Chassee T., Reeves M. (2018). Emergency dispatcher stroke recognition: Associations with downstream care. Prehosp. Emerg. Care.

[88] Jia J., Band R., Abboud M.E., Pajerowski W., Guo M., David G., Katz J.M., Newgard C.D., Callaway C.W., Pines J.M. (2017). Accuracy of emergency medical services dispatcher and crew diagnosis of stroke in clinical practice. Front. Neurol..

[89] Pottle J. (2019). Virtual reality and the transformation of medical education. Future Healthc. J..

[90] Bat-Erdene B.O., Saver J.L. (2021). Automatic acute stroke symptom detection and emergency medical systems alerting by Mobile health technologies: A review. J. Stroke Cerebrovasc. Dis..

[91] Kim H.S., Kim S.Y., Kim Y.H., Park K.S. (2015). A smartphone-based automatic diagnosis system for facial nerve palsy. Sensors.

[92] Raychev R.I., Saver J.L., Liebeskind D.S., Penkov S., Angelov D., Stoev K., Kim J., Chen Y., Liu X., Patel R. (2023). Abstract WMP120: Development of smartphone-enabled machine learning algorithms for autonomous stroke detection. Stroke.

[93] Figurell M.E., Meyer D.M., Perrinez E.S., Mills M.T., Shuey H.M., Wu D., Vyas R., Carroll J., Amin-Hanjani S., Almekhlafi M.A. (2023). Viz.ai implementation of stroke augmented intelligence and communications platform to improve indicators and outcomes for a comprehensive stroke center and network. AJNR Am. J. Neuroradiol..

[94] Gunda B., Neuhaus A., Sipos I., Toth G., Jovin T.G., Nogueira R.G., Mocco J., Fargen K.M., Liebeskind D.S., Yan B. (2022). Improved stroke care in a primary stroke center using AI-decision support. Cerebrovasc. Dis. Extra.

[95] Field N.C., Entezami P., Boulos A.S., Eker O., Heit J.J., Shakir H., Cruz-Gonzalez I., Jovin T.G., Nogueira R.G., Fargen K.M. (2023). Artificial intelligence improves transfer times and ischemic stroke workflow metrics. Interv. Neuroradiol..

[96] Devlin T., Collins G., Heath G.W., Majid A., Huang Y., Nogueira R.G., Jovin T.G., Fargen K.M., Menon B.K., Almekhlafi M.A. (2024). VALIDATE—Utilization of the Viz.ai mobile stroke care coordination platform to limit delays in LVO stroke diagnosis and endovascular treatment. Front. Stroke.

[97] Hassan A.E., Ringheanu V.M., Preston L., Alqaisi F., Fargen K.M., Nogueira R.G., Jovin T.G., Mocco J., Menon B.K., Zaidat O.O. (2022). Artificial intelligence–parallel stroke workflow tool improves reperfusion rates and door-in to puncture interval. Stroke Vasc. Interv. Neurol..

[98] Olive-Gadea M., Crespo C., Granes C., Hernandez-Perez M., Laredo C., Renard D., Gory B., Lapergue B., Blanc R., Piotin M. (2020). Deep Learning Based Software to Identify Large Vessel Occlusion on Noncontrast Computed Tomography. Stroke.

[99] McLouth J., Elstrott S., Chaibi Y., Toth G., Fraser J.F., Jovin T.G., Nogueira R.G., Jadhav A.P., Mocco J., Menon B.K. (2021). Validation of a deep learning tool in the detection of intracranial hemorrhage and large vessel occlusion. Front. Neurol..

[100] Yahav-Dovrat A., Saban M., Merhav G., Lankri I., Abergel E., Eran A., Tanne D., Nogueira R.G., Sivan-Hoffmann R. (2021). Evaluation of artificial intelligence-powered identification of large-vessel occlusions in a comprehensive stroke center. AJNR Am. J. Neuroradiol..

[101] Schlossman J., Ro D., Salehi S., Chow D., Yu W., Chang P.D., Soun J.E. (2022). Head-to-head comparison of commercial artificial intelligence solutions for detection of large vessel occlusion at a comprehensive stroke center. Front. Neurol..

[102] Luijten S.P.R., Wolff L., Duvekot M.H.C., van Doormaal P.J., Moudrous W., Kerkhoff H., Lycklama A.N., Bokkers R.P.H., Yo L.S.F., Hofmeijer J. (2022). Diagnostic performance of an algorithm for automated large vessel occlusion detection on CT angiography. J. NeuroInterventional Surg..

[103] Cimflova P., Golan R., Ospel J.M., Sojoudi A., Duszynski C., Elebute I., El-Hariri H., Hossein Mousavi S., Neto L.A.S.M., Pinky N. (2022). Validation of a machine learning software tool for automated large vessel occlusion detection in patients with suspected acute stroke. Neuroradiology.

[104] Bathla G., Durjoy D., Priya S., Samaniego E., Derdeyn C.P. (2022). Image-level detection of large vessel occlusion on 4D-CTA perfusion data using deep learning in acute stroke. J. Stroke Cerebrovasc. Dis..

[105] Adamou A., Beltsios E.T., Bania A., Katsanos A.H., Mavridis D., Mavridis K., Tsioufis C., Tsivgoulis G., Papadopoulos D., Tzoumas N. (2023). Artificial intelligence-driven aspects for the detection of early stroke changes in non-contrast CT: A systematic review and meta-analysis. J. NeuroInterventional Surg..

[106] Wolff L., Berkhemer O.A., Fransen P.S., van Zwam W.H., Lingsma H.F., van den Berg L.A., Beumer D., van Oostenbrugge R.J., Majoie C.B., Roos Y.B. (2021). Validation of automated Alberta Stroke Program Early CT Score (ASPECTS) software for detection of early ischemic changes on non-contrast brain CT scans. Neuroradiology.

[107] Lee S.J., Park G., Kim D., Jung S., Song S., Hon J.M., Shin D.H., Lee J.S. (2024). Clinical evaluation of a deep-learning model for automatic scoring of the Alberta Stroke Program Early CT Score on non-contrast CT. J. NeuroInterventional Surg..

[108] Jiang B., Pham N., van Staalduinen E.K., Tien S., Chen H., Wang Y., Li C., Zhang D., Chen X., Liu F. (2025). Deep learning applications in imaging of acute ischemic stroke: A systematic review and narrative summary. Radiology.

[109] Alwood B.T., Meyer D., Inonita C., Bhogal P., McDonough R., Goyal M., Menon B.K., Demchuk A.M., Liebeskind D.S., Jahan R. (2024). Multicenter comparison using two AI stroke CT perfusion software packages for determining thrombectomy eligibility. J. Stroke Cerebrovasc. Dis..

[110] Ali F., Hamid U., Zaidat O., Qureshi A.I., Jadhav A.P., Fargen K.M., Nogueira R.G., Mocco J., Jovin T.G., Menon B.K. (2020). Role of artificial intelligence in telestroke: An overview. Front. Neurol..

[111] Mohamed A., Elsherif S., Legere B., Seetharam K., Shuaib A., Shuaib W., Dineen R.A., Jayaraman M.V., Jahan R., Almekhlafi M.A. (2023). Is telestroke more effective than conventional treatment for acute ischemic stroke? A systematic review and meta-analysis of patient outcomes and thrombolysis rates. Int. J. Stroke.

[112] Vélez-Guerrero M.A., Callejas-Cuervo M., Mazzoleni S. (2021). Artificial intelligence-based wearable robotic exoskeletons for upper limb rehabilitation: A review. Sensors.

[113] Várkuti B., Guan C., Pan Y., Pham M., Leeb R., Toni I., Millán J.d.R., Birbaumer N., Bütefisch C.M. (2012). Resting state changes in functional connectivity correlate with movement recovery for BCI and robot-assisted upper-extremity training after stroke. Neurorehabil Neural Repair..

[114] Cervera M.A., Soekadar S.R., Ushiba J., Millán J.d.R., Liu M., Birbaumer N., Cohen L.G., Ramos-Murguialday A. (2018). Brain-computer interfaces for post-stroke motor rehabilitation: A meta-analysis. Ann. Clin. Transl. Neurol..

[115] Bundy D.T., Souders L., Baranyai K., Mrachacz-Kersting N., Frolov A., Guan C., Várkuti B., Kraus D., Burianová J., Cervera M.A. (2017). Contralesional brain–computer interface control of a powered exoskeleton for motor recovery in chronic stroke survivors. Stroke.

[116] Mrachacz-Kersting N., Jiang N., Stevenson A.J., Farina D., Dremstrup K., Burianová J., Kraus D., Várkuti B., Cervera M.A., Guan C. (2016). Efficient neuroplasticity induction in chronic stroke patients by an associative brain-computer interface. J. Neurophysiol..

[117] Senadheera I., Hettiarachchi P., Haslam B., Jayawardena S., Bandara T., Perera H., de Silva D., Fernando S., Pathirana P., Gunawardena S. (2024). AI applications in adult stroke recovery and rehabilitation: A scoping review using AI. Sensors.

[118] Bütefisch C.M. (2015). Role of the contralesional hemisphere in post-stroke recovery of upper extremity motor function. Front. Neurol..

[119] Dobkin A. (2024). A Scoping Review of AI Applications in Adult Stroke Recovery and Rehabilitation. Int. J. Neurorehabilit..

[120] Choo Y.J., Chang M.C. (2022). Use of machine learning in stroke rehabilitation: A narrative review. Brain Neurorehabilit..

[121] Sale P., Ferriero G., Ciabattoni L., Cortese A.M., Ferracuti F., Romeo L., Piccione F., Masiero S. (2018). Predicting Motor and Cognitive Improvement Through Machine Learning Algorithm in Human Subject that Underwent a Rehabilitation Treatment in the Early Stage of Stroke. J. Stroke Cerebrovasc. Dis..

[122] Kim S., Park S.W., Jeong T., Lee J.H., Choi Y., Lim J., Han K., Jung H., Oh M., Kang H. (2025). AI-driven cognitive telerehabilitation for stroke: A randomized controlled trial. Front. Neurol..

[123] Liscano Y., Bernal L.M., Rodríguez M., Diaz Vallejo J.A. (2025). Effectiveness of AI-assisted digital therapies for post-stroke aphasia rehabilitation: A systematic review. Brain Sci..

[124] Kim W., Cho S., Baek D., Bang H., Paik N. (2016). Upper extremity functional evaluation by fugl-meyer assessment scoring using depth-sensing camera in hemiplegic stroke patients. PLoS ONE.

[125] Shull P.B., Jiang S., Zhu Y., Zhu X. (2019). Hand gesture recognition and finger angle estimation via wrist-worn modified barometric pressure sensing. IEEE Trans. Neural Syst. Rehabil. Eng..

[126] Fassbender K., Walter S., Grunwald I.Q., Merzou F., Mathur S., Lesmeister M., Kappel A., Weber R., Müller C., Schmidt D. (2020). Prehospital stroke management in the thrombectomy era. Lancet Neurol..

[127] Walsh K.B. (2019). Non-invasive sensor technology for prehospital stroke diagnosis: Current status and future directions. Int. J. Stroke.

[128] Schwartz J., Dreyer R.P., Murugiah K., Ranasinghe I. (2016). Contemporary prehospital emergency medical services response times for suspected stroke in the United States. Prehosp. Emerg. Care.

[129] Kellner C.P., Sauvageau E., Snyder K.V., Fargen K.M., Arthur A.S., Turner R.D., Kan P., Levy E.I., Mocco J., Rasmussen P.A. (2018). The VITAL study and overall pooled analysis with the VIPS non-invasive stroke detection device. J. NeuroInterventional Surg..

[130] Ebinger M., Siegerink B., Kunz A., Wendt M., Weber J.E., Schwabauer E., Malzahn U., Heuschmann P.U., Fiebach J.B., Endres M. (2021). Association between dispatch of mobile stroke units and functional outcomes among patients with acute ischemic stroke in Berlin. JAMA.

[131] English S.W., Barrett K.M., Freeman W.D., Demaerschalk B.M. (2022). Telemedicine-enabled ambulances and mobile stroke units for prehospital stroke management. J. Telemed. Telecare.

[132] Shlobin N.A., Baig A.A., Waqas M., Rai A.T., Turk A.S., Siddiqui A.H., Levy E.I., Mocco J., Nogueira R.G., Zaidat O.O. (2022). Artificial intelligence for large-vessel-occlusion stroke: A systematic review. World Neurosurg..

[133] Viz.ai (2018). Viz.ai Granted De Novo FDA Clearance for First Artificial Intelligence Triage Software.

[134] RapidAI (2020). RapidAI Receives FDA Clearance of Rapid LVO.

[135] Brainomix (2023). Brainomix Receives FDA Clearance for Its Flagship Stroke AI Imaging Software (e-ASPECTS).

[136] Avicenna.AI (2024). Avicenna.AI Secures FDA Clearance for Two Healthcare AI Solutions Including CINA-ASPECTS.

[137] Aidoc (2022). Aidoc Secures FDA Clearance for AI Stroke Software Detecting Large Vessel Occlusion on CTA.

[138] Methinks Software (2025). FDA Clears AI Software Tool for Automated Detection of LVOs from CT Angiography (Methinks CTA Stroke).

[139] Iosa M., Paolucci S., Antonucci G., Ciancarelli I., Morone G. (2023). Application of an Artificial Neural Network to Identify the Factors Influencing Neurorehabilitation Outcomes of Patients with Ischemic Stroke Treated with Thrombolysis. Biomolecules.

[140] Campagnini S., Arienti C., Patrini M., Liuzzi P., Mannini A., Carrozza M.C. (2022). Machine learning methods for functional recovery prediction and prognosis in post-stroke rehabilitation: A systematic review. J. Neuroeng. Rehabil..

[141] Nishi H., Oishi N., Ishii A., Ono I., Ogura T., Sunohara T., Chihara H., Fukumitsu R., Okawa M., Yamana N. (2019). Predicting Clinical Outcomes of Large Vessel Occlusion Before Mechanical Thrombectomy Using Machine Learning. Stroke.

[142] Al-Janabi O.M., El Refaei A., Elgazzar T., Mahmood Y.M., Bakir D., Gajjar A., Alateya A., Jha S.K., Ghozy S., Kallmes D.F. (2024). Current Stroke Solutions Using Artificial Intelligence: A Review of the Literature. Brain Sci..

[143] Powers W.J., Rabinstein A.A., Ackerson T., Adeoye O.M., Bambakidis N.C., Becker K., Biller J., Brown M., Demaerschalk B.M., Hoh B. (2019). Guidelines for the early management of patients with acute ischemic stroke: 2019 update to the 2018 guidelines for the early management of acute ischemic stroke: A guideline for healthcare professionals from the American Heart Association/American Stroke Association. Stroke.

[144] Budhu J.A., Anderson N., Branson C., Choudhury A., Du R., Essig M., Garg N., Gaynor E., Greenberg B., Johnson C. (2025). Health equity considerations in the age of artificial intelligence. Neurology.

